# Phase II study of erlotinib (OSI-774) in patients with metastatic colorectal cancer

**DOI:** 10.1038/sj.bjc.6603055

**Published:** 2006-03-28

**Authors:** C A Townsley, P Major, L L Siu, J Dancey, E Chen, G R Pond, T Nicklee, J Ho, D Hedley, M Tsao, M J Moore, A M Oza

**Affiliations:** 1 Princess Margaret Hospital Phase II Consortium, Department of Medical Oncology and Hematology, Princess Margaret Hospital, University of Health Network, University of Toronto, 610 University Avenue, Toronto, Ontario, Canada M5G 2M9; 2 Cancer Therapy Evaluation Program, National Cancer Institute, Rockville, MD, USA

**Keywords:** clinical trial, erlotinib, colorectal cancer, EGFR, correlative markers, phase II

## Abstract

Erlotinib (Tarceva™, OSI-774), a potent epidermal growth factor receptor tyrosine kinase inhibitor (EGFR), was evaluated in a phase II study to assess its activity in patients with metastatic colorectal cancer. In all, 38 patients with metastatic colorectal cancer were treated with erlotinib at a continuous daily oral dose of 150 mg. Radiological evaluation was carried out every 8 weeks and tumour biopsies were performed before treatment and on day 8. Of 31 evaluable patients, 19 (61%) had progressive disease and 12 (39%) had stable disease (s.d.). The median time to progression for those patients having s.d. was 123 days (range 108–329 days). The most common adverse events were rash in 34 patients and diarrhoea in 23 patients. Correlative studies were conducted to investigate the effect of erlotinib on downstream signalling. Tumour tissue correlations were based on usable tissue from eight match paired tumour samples pre- and on therapy, and showed a statistically significant decrease in the median intensity of both pEGFR (*P*=0.008) and phospho-extracellular signal-regulated kinase (ERK) (*P*=0.008) a week after commencement of treatment. No other statistically significant change in tumour markers was observed. Erlotinib was well tolerated with the most common toxicities being rash and diarrhoea. More than one-third of evaluable patients had s.d. for a minimum of 8 weeks. Correlative studies showed a reduction in phosphorylated EGFR and ERK in tumour tissue post-treatment.

Colorectal cancer is one of the most common malignancies in North America, with an annual incidence around 150 000 and mortality of 60 000 per year (Ries *et al*, 1999). Although there have been many significant advances in the treatment of metastatic colon cancer over the past decade with combination chemotherapy and sequential regimens incorporating fluoropyrimidines, irinotecan, oxaliplatin and newer biological agents, the median survival remains between 20 and 22 months for patients with metastatic disease (Giacchetti *et al*, 2000; Saltz *et al*, 2000).

Molecular targeted agents that specifically modulate inherent abnormalities in malignant cells have opened new treatment possibilities. The epidermal growth factor receptor (EGFR) is a transmembrane glycoprotein, which, once activated by a growth stimulus, initiates a signal transduction cascade of biochemical, and physiological changes that culminate in mitogenic signalling and other tumour-promoting cellular activities (Carpenter and Cohen, 1990). There are compelling data that dysregulation of this transduction pathway plays an important role in the process of malignant transformation, metastasis and resistance to apoptosis (Ullrich and Schlessinger, 1990). It has also been shown that mutant EGFRs selectively activate signalling pathways that promote cell survival (Salomon *et al*, 1995).

Epidermal growth factor receptor is overexpressed in many different cancer types, including 60–80% of colon cancers (Porebska *et al*, 2000), which correlates with a poor prognosis (Mayer *et al*, 1993). This further supports the hypothesis that inhibition of the EGFR signalling cascade may potentially be therapeutic. Clinical trials using EGFR inhibitors such as Cetuximab (IMC-C225) have shown encouraging activity both as a monotherapy as well as in combination with either chemotherapy or radiation, including patients with head and neck squamous cell carcinoma, non-small-cell lung cancer and colorectal cancer (Rubin *et al*, 2000; Saltz *et al*, 2002). Cetuximab was clinically effective in patients with irinotecan-resistant colon cancer both when given alone and in combination with irinotecan, with a response rate of 22.9% and survival of 8.6 months in the combination group while the monotherapy group had a response rate of 10.8% and a survival of 6.9 months (Cunningham *et al*, 2004).

Erlotinib hydrochloride, [6,7-Bis(2-methoxy-ethoxy)-quinazolin-4-yl]-(3-ethynyl-phenyl) amine hydrochloride, also known as CP-358,774, OSI-774 and Tarceva™, is an orally active potent selective inhibitor of the EGFR tyrosine kinase (TK). It competes with the ATP-binding site in the intracellular TK domain of EGFR with an IC_50_ of 2 nM against the kinase. Erlotinib induces apoptosis in selective, *in vitro* tumour cell lines and has antiproliferative activity against numerous human tumour xenografts *in vivo* (Pollack *et al*, 1999). In clinical trials, erlotinib has shown antitumour activity in several malignancies, including lung, pancreas, ovarian, head and neck, endometrial and biliary tract cancers (Jasas *et al*, 2004; Perez-Soler *et al*, 2004; Philip *et al*, 2004; Soulieres *et al*, 2004; Gordon *et al*, 2005). It has been found to be safe and well tolerated with the most common side effects being diarrhoea, rash, nausea, headache, emesis and fatigue. The recommended phase II dose is 150 mg orally per day on a continuous schedule.

This phase II study of erlotinib in patients with metastatic colorectal cancer was conducted to evaluate its efficacy as a single agent. The objectives of the study were (1) to assess the efficacy (response rate and duration of stable disease (s.d.)) of erlotinib given daily in patients with recurrent or metastatic colorectal cancer, (2) to assess the toxicity, time to progression and (3) to investigate the effect of erlotinib on EGFR inhibition and downstream signalling pathways.

## PATIENTS AND METHODS

### Patient eligibility

The research ethics board at each participating institution approved the protocol and all patients enrolled on study gave written informed consent in accordance with federal and institutional guidelines before treatment. Patients were eligible to enter the study if they had histologically or cytologically confirmed metastatic adenocarcinoma of the colon or rectum. Patients could not have had more than one line of chemotherapy for metastatic disease, not including adjuvant chemotherapy. However, patients who were treated with 5-fluorouracil followed by irinotecan separately for advanced disease were still eligible. Participants were required to have an Eastern Cooperative Oncology Group (ECOG) performance status of 0 to 2, age greater than or equal to 18 years and a life expectancy of at least 3 months. Any patients requiring anticoagulation were put on heparin instead of coumadin owing to the potential for bleeding when erlotinib is used in conjunction with coumadin. Patients were required to have radiographically measurable disease.

Patients had to have completed any previous chemotherapy at least 4 weeks before the planned start of erlotinib. Prior radiation was allowed, provided the last fraction of radiotherapy was more than 4 weeks from the planned start of erlotinib. Patients had to have normal organ and marrow function as follows: absolute granulocyte count ⩾1.5 × 10^9^ l^−1^, platelets ⩾100 000 × 10^9^ l^−1^, total bilirubin and serum bilirubin ⩽1.25 × upper normal limit (UNL) aspartate aminotransferase (AST) serum glutamic-oxaloacetic transaminase (SGOT) or alanine aminotransferase (ALT) serum glutamic-pyruvic transaminase (SGPT) ⩽3 × UNL (⩽5 × in the presence of liver metastases) and creatinine ⩽1.25 × UNL or a calculated creatinine clearance ⩾50 ml min^−1^. Patients had to be willing and able to undergo tumour biopsy once before and once during therapy. In cases where there was a medical contraindication to tumour biopsy, exemption was granted after discussion with the Principal Investigator, if appropriate. Patients who required therapeutic doses of warfarin were changed to low molecular weight heparin at least 3 days before starting erlotinib, if this was considered medically acceptable; if not, patients were excluded from the study. Before the activation of the study, all centres obtained approval of their respective institutional ethics review boards.

### Study design and treatment plan

All treatment was delivered in an outpatient setting. Erlotinib was administered at a continuous, daily dose of 150 mg. Patients were instructed to take erlotinib with at least 200 ml of water each morning before breakfast, and they had to fast for at least 2 h before and 1 h after treatment. A cycle was considered to be 28 days. Before starting study medication and on day 8 of therapy, patients underwent tumour biopsies, unless medically contraindicated. Tumour biopsies were performed as either ultrasound-guided liver core needle biopsies or tissue biopsies performed during a colonoscopic procedure if possible.

Treatment was continued until one of the following criteria was met: (1) disease progression, (2) intercurrent illness that prevented further administration of treatment, (3) unacceptable adverse event(s), (4) patient's decision to withdraw from the study or (5) general or specific changes in the patient's condition render the patient unacceptable for further treatment in the judgment of the investigator.

### On-study evaluation

Computerised tomography was used for radiological evaluation and was performed at baseline and after every two cycles or 8 weeks. It was also performed 4 weeks after any response or s.d. was sent to confirm that end point, as well as at any time when there was clinical suspicion of progressive disease. Tumour responses were categorised as a complete or partial response, or progressive disease or s.d. Response and progression were defined by the Response Evaluation Criteria in Solid Tumours (RECIST) (Therasse *et al*, 2000). Stable disease was defined as any change in tumour size that did not meet the criteria for progressive disease or partial response at 8 weeks and that persisted for a minimum of an additional 4 weeks. The best overall response was the best response recorded from the start of the treatment until disease progression. The duration of response was defined as the time from complete or partial response or s.d. until the first objective evidence of disease progression or death from any cause. Time to progression was defined as the time from enrolment onto the study until progression or death. Toxicities were evaluated on days 1 and 15 of each cycle and graded according to the National Cancer Institute common toxicity criteria version 2.0.

The treatment of the erlotinib-related skin rash was as follows: if skin rash was mild or grade 1, a topical silver sulphadiazine cream (flamazine® 1%) was applied twice daily to affected areas. For more severe or persistent skin rash (grade 2 or higher), a course of oral minocycline was prescribed (200 mg per os (p.o.) as a loading dose, followed by 100 mg p.o. twice daily (b.i.d.) × 7–10 days). Patients with diarrhoea were treated with loperamide 4 mg at first onset, followed by 2 mg q 2–4 h until diarrhoea free for 12 h. Patients with grade 3 or 4 skin rash or diarrhoea were given 1 week off drug therapy to evaluate whether the rash or diarrhoea would resolve. Patients whose rash or diarrhoea did not return to a grade 1 or less within 2 weeks were taken off study.

### Pharmacokinetics

Blood samples were collected from a peripheral vein in 7 ml heparinised tubes at baseline and before drug administration on day 8, day 15, day 29 and day 57 of therapy. The specimens were centrifuged at 3000 **g** for 15 min at room temperature. Plasma samples were stored at −80°C until analysis. Erlotinib and OSI-420 concentrations were quantified with a sensitive and specific high-pressure liquid chromography method (Zhang *et al*, 2005).

### Immunohistochemistry

Core needle tumour biopsies were taken before and after 1 week of treatment with erlotinib. They were collected into 10% neutral-buffered formalin, fixed overnight before transferred to 70% ethanol and shipped to Princess Margaret Hospital for processing into paraffin blocks. Sections of 4 *μ*m thickness was cut onto Surgipath x-tra slides with pretreatment and on-treatment specimen placed side by side on the same slide. Sections were dewaxed in xylene and rehydrated through graded alcohol. Epidermal growth factor receptor status was determined on sections cut from archival paraffin blocks after pretreatment in 0.4% pepsin, pH 2.0, and stained for immunohistochemistry with EGFR monoclonal antibody (Zymed, Markham, Ontario, Canada; clone 31G7, 1 : 50 dilution). Antigen retrieval was performed on the other slides in a Milestone T/T Mega microwave oven for 10 min at 120°C in 10 mM citrate buffer, pH 6.0. After cooling, sections were covered with normal serum and then incubated in primary antibodies. Tumour biopsies were stained using immunofluorescence techniques and dual labelling for cytokeratin (cocktail of Dako monoclonal antibodies to AE1/AE3, HMWK, CK7, CK20 or Dako rabbit polyclonal antikeratin wide spectrum screening) was usually carried out to help with the identification of epithelial tumour cells. Slides were rinsed in phosphate-buffered saline (PBS) and the appropriate secondary antibodies (Cy3 conjugated for the pharmacodynamic markers and Cy5 conjugated for cytokeratin) were applied for 30 min at room temperature in darkness. After rinsing in PBS, sections were counterstained with the DNA-specific dye 4,6-diamidino-2-phenylindole (DAPI; Roche, Indianapolis, IN, USA) at 1 *μ*g ml^−1^ for 5 min at 4°C, then rinsed twice for 5 min with distilled water and allowed to air dry. Slides were stored in the refrigerator until image analysis was performed.

### Correlative analysis

Archival paraffin blocks were acquired to determine the EGFR status of the patient at the time of diagnosis. A pathologist reviewed sections stained for immunohistochemistry with EGFR, using a transmitted light microscope and scored the percent positive staining tumour cells. The haematoxylin and eosin (H&E) sections were scanned using a Polaroid SprintScan 35 Plus and a PathScan Enabler (Meyer Instruments, Houston, TX, USA). Variable amounts of necrotic and normal tissues were interspersed with tumour tissue. Colour printouts of the entire biopsy were then used to map the areas of viable tumour tissues for analysis in the fluorescence-stained sections.

Serial sections that had been immunofluorescent stained for the above antibodies were then imaged and analysed using the MicroComputer Image Device software, Elite version 6 (Imaging Research, St Catharines, ON, Canada) interfaced with a Quantix Cooled CCD camera (Photometrics, Tucson, AZ, USA) and a Ludl Biopoint motorised stage (Ludl Electronic Products, Hawthorne, NY, USA) both affixed an Olympus BX50 reflected fluorescence microscope (Olympus, Melville, NY, USA). For antibodies MIB-1, p27, UPlanFl × 20 objective lens with numeric aperture of 0.50 was used. The remaining antibodies phospho-protein kinase B (PKB)/AKT, extracellular signal-regulated kinase (ERK), phospho-ERK, EGFR, phospho-EGFR, were imaged using UPlanFl × 10 objective lens with numeric aperture of 0.30. MicroComputer Image Device coordinated the generation of a digitised field-by-field tiled image of the selected tumour area.

Images for each fluorochrome were sequentially acquired. To measure the nuclear levels of p27 and Ki67/MIB-1, DAPI image was used to create a nuclear mask, from which the total nuclear count was made. Then, nuclear mask was overlaid on the Cy3 fluorescence Ki67/MIB-1 and p27 images. By setting the appropriate threshold, only positive Ki67/MIB-1 and p27 nuclei were counted, yielding a labelling index.

Using cytokeratin-positive areas to outline corresponding tumour tissue, the mean integrated optical densities for signalling proteins, EGFR, phospho-EGFR, ERK, phospho-ERK, PKB/Akt, phospho-PKB/Akt and p27 was measured. Negative control slides from each biopsy were run in parallel. The primary antibody was omitted, but cytokeratin was still used to identify areas of tumour. The mean optical density from the control slides was used to obtain a baseline of endogenous fluorescence. This value was then subtracted from the matching mean integrated optical density of the test slides.

### Statistical methods

The primary end point was objective response or disease stabilisation. The study design was based on the multinomial end-point design as in Zee *et al* (1999) and allowed for early study termination after 15 patients if (1) one or less responses and 11 or more early progressions, or (2) 12 or more early progressions were observed. Of the first 15 evaluable patients, five patients had s.d. and 10 had early progression, thus, although no responses were observed, the early stopping rule was not met and accrual continued to stage 2. After 30 patients were accrued, to accept the drug as active, we required (1) one or more responses and 19 or less early progressions; (2) three or more responses and 20 or less early progressions or (3) four or more responses and 22 or less early progressions. This was based on a test with null hypothesis being that the response rate was 5% and the early progression rate was 80% *vs* the alternative hypothesis that the response rate was 20% and the early progression rate was 60% and having significance level of 0.049 and 82% power.

Summary statistics, such as the mean, median and frequency, were used to describe the characteristics of the patients enrolled to this study. The Kaplan–Meier method was used to estimate overall and progression-free survival. Demographic and adverse event information was collected for all patients receiving at least one dose of treatment. Response and follow-up information was collected on all eligible patients. All patients with available tumour biopsies pre- and on-treatment were included in the immunohistochemistry and quantitative image analyses.

Changes in immunohistochemistry and quantitative image measurement values from pre- to on-treatment were evaluated using the Wilcoxon signed-rank test. Differences in baseline value and the change in value (pre- to on-treatment) for immunohistochemistry and quantitative image measurements between patients grouped by response status (patients with s.d. ⩾8 weeks *vs* patients with disease progression <8 weeks) were compared using the Wilcoxon rank-sum test.

All tests were two-sided and a *P*-value of less than 0.05 was considered statistically significant. Exact *P*-values are provided for all statistical tests. All statistical calculations were performed in SAS v.8 (SAS Institute, Cary, NC, USA) and figures plotted in S-plus 2000 (Insightful Corp., Seattle, WA, USA).

This study was supported through a National Cancer Institute/Cancer Therapy Evaluation Program grant.

## RESULTS

### Patient characteristics

A total of 38 patients were enrolled onto the study. The median age was 63.9 (range 40.9–78.1), 25 (66%) were male, 16 (42%) patients had received prior adjuvant chemotherapy, 32 (84%) had received chemotherapy for metastatic disease and 12 (31%) patients had received prior radiotherapy. Patient demographics are summarised in Table 1.

### Treatment administration

A total of 112 complete or partial cycles were administered, with a median of two cycles (range 1–7 cycles). Seven patients required at least one dose reduction because of rash or diarrhoea and seven patients required treatment delays because or rash or diarrhoea.

### Objective response

Of 38 patients enrolled on this study, seven patients were not evaluable for response because they were discontinued from the study in cycle 1, for a variety of reasons. One patient each developed cord compression/brain metastases during cycle 1, bowel obstruction on day 7 of cycle 1, noncompliance after day 22 of cycle 1 for toxicity, noncompliance after day 28 of cycle 1 for reasons not stated, symptomatic progression after day 18, grade 3 vomiting after day 18 of cycle 1 and disease progression on day 23. Of the 31 evaluable patients, 19 (61%) had progressive disease and 12 (39%) had s.d. If the seven inevaluable patients are included, the intent-to-treat analysis demonstrates a s.d. rate of 32.6% (12 of 38). Patients with s.d. had a median duration of five cycles. Five patients with s.d. completed six cycles, two patients with s.d. completed five cycles and five patients with s.d. completed four cycles of therapy. There were no complete or partial responses seen.

### Time to disease progression

The median number of days until clinical progression was 56 (range 53–112) days and median number of days until progression for those patients having s.d. is 123 (range 108–329) days.

### Survival

**Figure 1:**
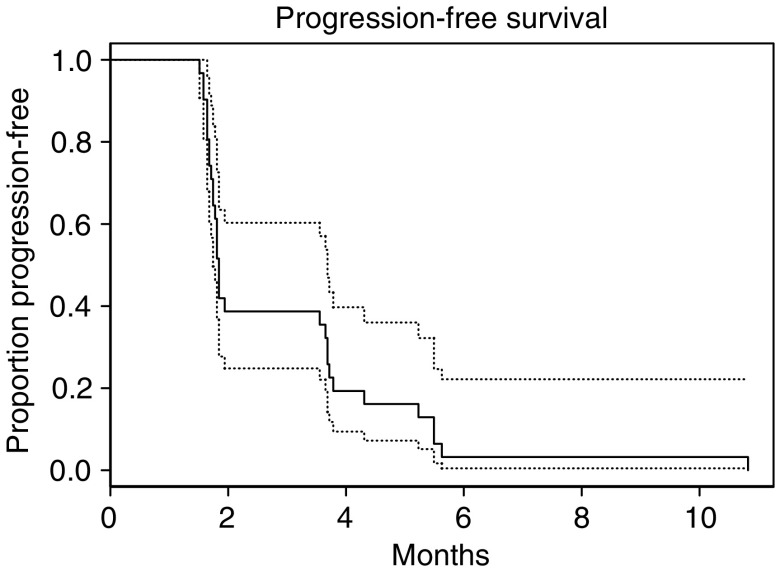
Progression-free survival of patients on this phase II study of erlotininb with 95% CI.

**Figure 2:**
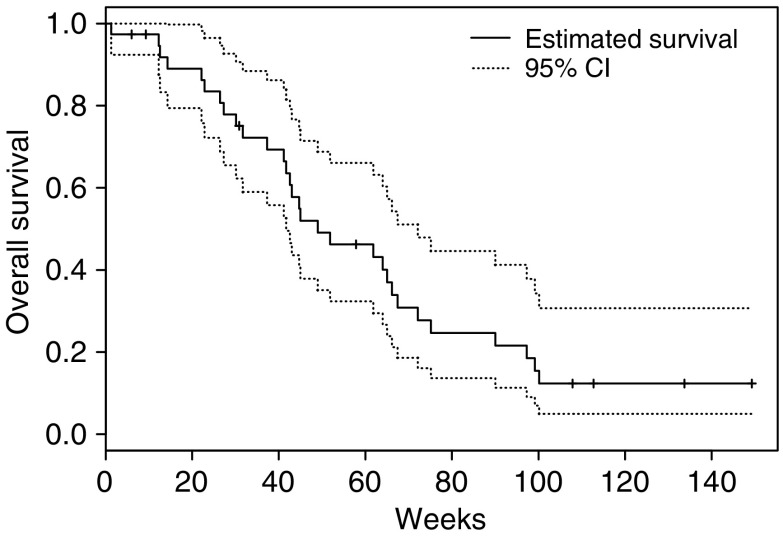
Overall survival, as of December 2004, of patients on this phase II study of erlotinib in weeks with 95% CI.

The 6-month survival was 83.5% (95% confidence interval (CI) 72.2, 96.5%), the 12-month survival was 46.2% (95% CI 32.3, 66.1%), the 18-month survival was 24.7% (95% CI 13.6, 44.6%) and the median survival was 11.3 months (95% CI 9.6, 16.6). Progression-free survival and overall survival are shown in Figures 1 and 2, respectively.

### Toxicity

The most common toxicity was rash in 34 patients over 88 cycles (79% of cycles), diarrhoea in 23 patients over 69 cycles (62%), lymphopenia in 33 patients over 81 cycles (72%), fatigue/lethargy in 24 patients over 63 cycles (56%) and hyperglycaemia in 23 patients over 69 cycles (62%). There were two fatal adverse events. One patient developed spinal cord compression, and was found to have brain metastases, during cycle 1. Another patient developed a bowel obstruction on day 7 of cycle 1. Both events were felt to be unlikely related to erlotinib. There were 45 grade 3 or 4 adverse events experienced, including grade 4 constipation in two patients, grade 4 abdominal pain/cramping, grade 4 bone (left hip and right ankle) pain and grade 3 pain in two patients (right flank for two cycles, left flank), grade 3 rash in four patients, grade 3 diarrhoea in four patients and grade 3 vomiting in three patients. Adverse events were summarised in Tables 2 and 3.

### Pharmacokinetics

**Figure 3:**
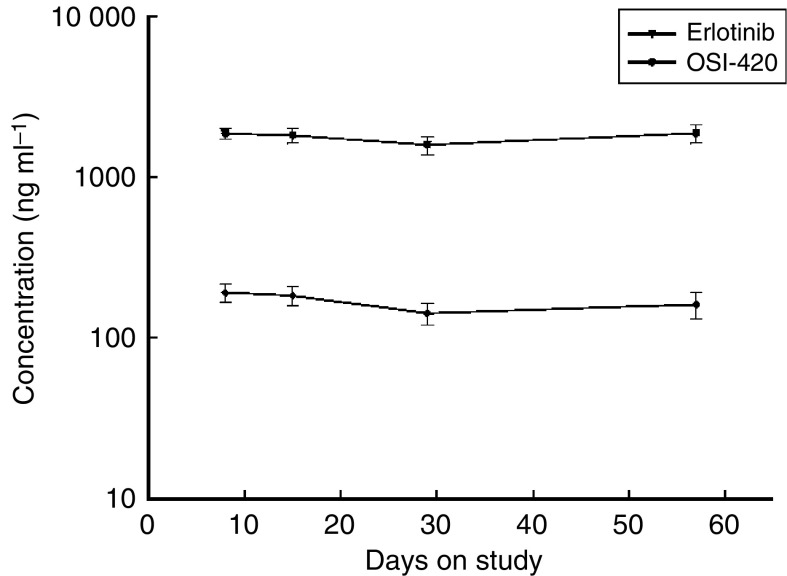
Pharmacokinetic analysis of erlotinib and its metabolite.

**Figure 4:**
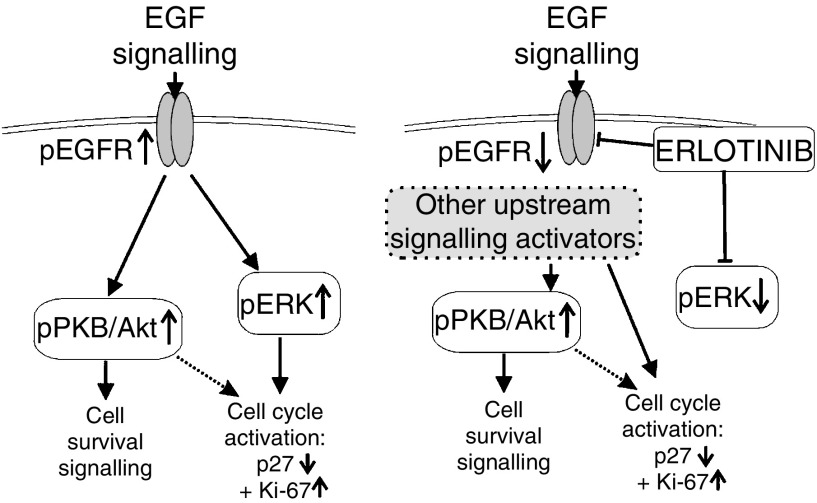
EGFR signalling pathway, including downstream markers and the proposed effects of erlotinib.

The mean minimum steady-state concentrations ((Css)min) of erlotinib and OSI-420 were shown in Figure 3. The (Css)min of erlotinib is above the IC_50_, indicating that sufficiently high concentration of erlotinib for target inhibition could be achieved at this dose level (Figure 4).

### Correlative studies

#### Tumour biopsies

**Figure 5:**
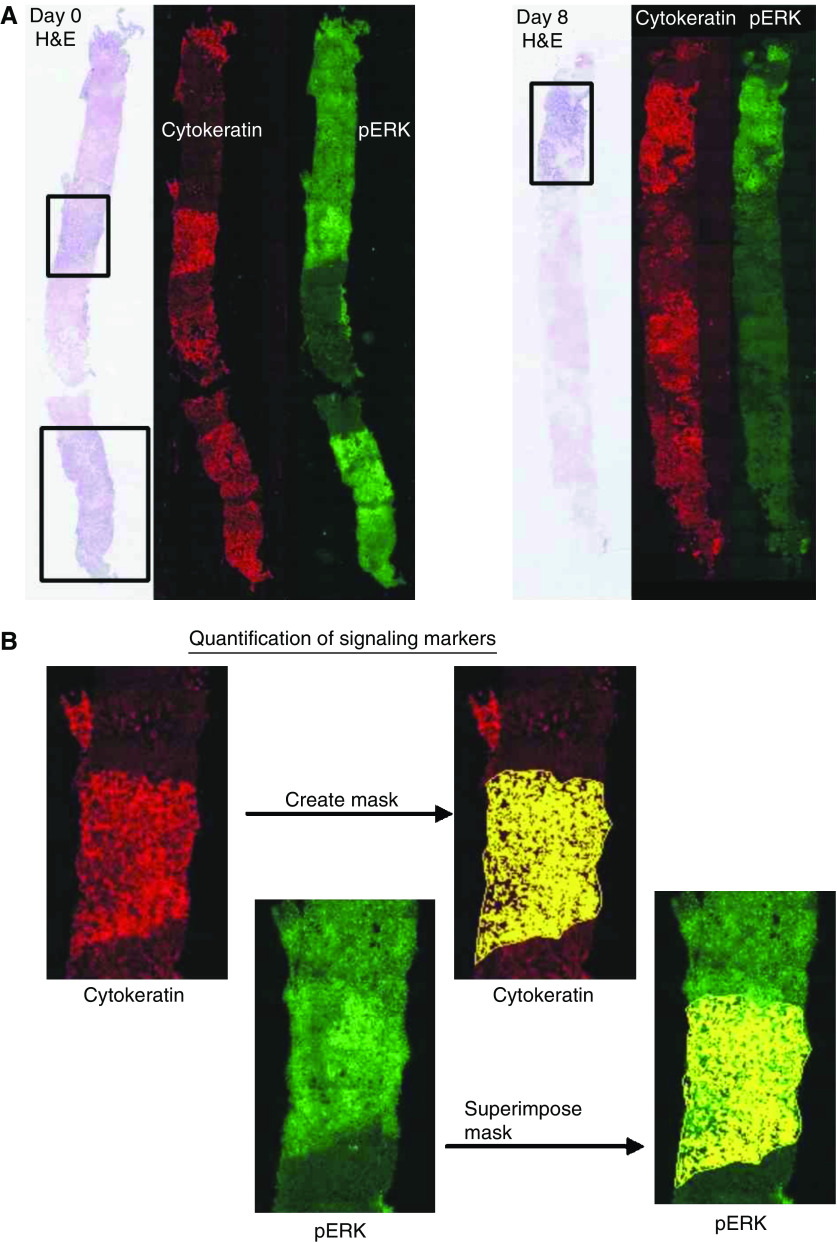
(**A**) Core biopsies obtained from liver metastases pretreatment (left panels) and post-treatment (right panels), showing haematoxylin and eosin staining by transmitted light, and dual fluorescence staining for cytokeratin (red) and phosphorylated ERK1/2 (green). The fluorescence images have been false coloured and contrast enhanced for visual inspection. (**B**) Image processing used to quantify phosphospecific antibody labelling. The images were analysed by first converting the cytokeratin image into a binary (false coloured yellow), and overlaying this onto the linked phosphorylated protein image in order to outline the tumour area. After background subtraction, the mean pixel grey-scale values within these areas were used as a measurement of the extent of phosphorylated protein expression.

Figure 5 illustrates the use of wide field, dual colour fluorescence image analysis to measure erlotinib pharmacodynamic effects in pre- and post-treatment liver biopsies. Using the H&E-stained section as a guide and the ELITE image analysis software, cytokeratin-positive tumour areas were outlined. By setting a threshold on the cytokeratin staining, a mask of the tumour area was created. This mask was then superimposed on the corresponding Cy3 image for signalling proteins, EGFR, phospho-EGFR, ERK, phospho-ERK, PKB/Akt, phospho-PKB/Akt and p27. The software then calculated the mean integrated optical density under the mask. Negative control slides from each biopsy were run in parallel. The primary antibody was omitted, but cytokeratin was still used to identify areas of tumour. The mean optical density from the control slides was used to obtain a baseline of endogenous fluorescence. This value was then subtracted from the matching mean integrated optical density of the test slides.

**Figure 6:**
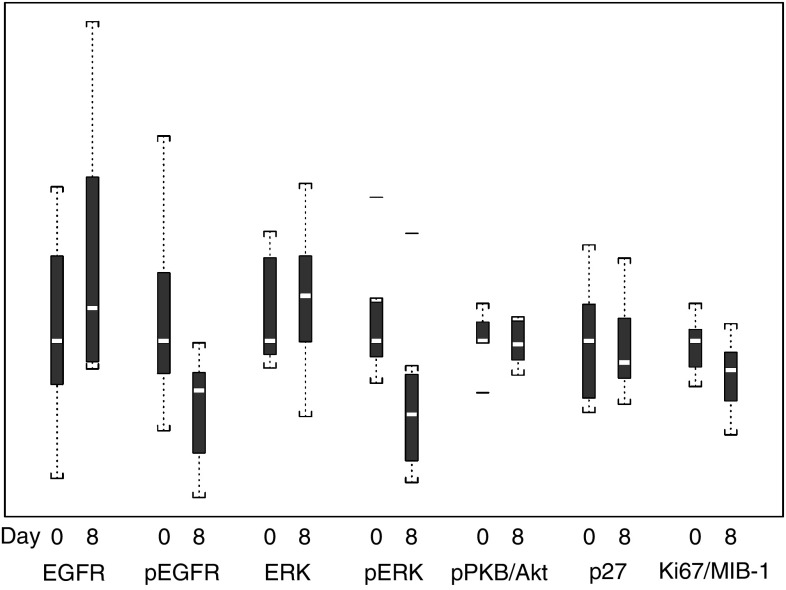
Whisker plot of change in markers pre- and on-treatment.

Owing to problems with degradation of tissues during storage and transportation as well as medical contraindications to biopsies in some patients, only eight patients had matched biopsies from prestudy and after day 7 that were analysable. Results were summarised in Table 4 and Figure 6. There were significant decreases in the levels of phosphorylated EGFR (*P*<0.01) and phosphorylated ERK (*P*<0.01; Wilcoxon's signed–rank test), with no significant changes in the total levels of these proteins. There was no significant change in the level of phosphorylated PKB/Akt, or in the proliferation-associated markers p27 and Ki67/MIB-1.

Figure 4 demonstrates the pathway of the EGFR downstream markers that were evaluated in this study. The left side of the diagram illustrates the effects of EGF signalling on downstream markers. The right side of the diagram shows the hypothesised effects of erlotinib to as a possible explanation for results observed in this study. It is possible that while erlotinib has direct inhibitory effects on phospho-EGFR and phospho-ERK, other upstream signalling activators maintain activation of the downstream cascade.

### Pharmacodynamics

Although there were no responses observed in this study, changes in correlative markers were analysed to determine if there was a correlation with s.d. The change in phospho-ERK from pretreatment to on-treatment was not statistically significantly associated with clinical outcome (*P*-value=0.34), similarly the change in phospho-EGFR was not associated with clinical outcome (*P*-value=0.53).

## DISCUSSION

There is a strong rationale for targeting the EGFR family for cancer treatment: expression/overexpression is frequently seen in carcinomas and brain tumours; overexpression has been shown to correlate with poor outcome in some studies; aberrant signalling through EGFR family is tumorigenic and pharmacological inhibition is associated with tumour inhibition in multiple nonclinical models. There are currently many antibodies and small molecules that target the EGFR family under different phases of development; some of which, such as cetuximab, are showing promising clinical activity in patients with advanced colorectal carcinoma.

This phase II study examined the efficacy and safety of erlotinib, an oral EGFR TK inhibitor, in patients with metastatic colorectal cancer. Although there were no observed responses, an s.d. rate of 39% for a median of 4 months was interesting in a population that had already been treated with at least one line of chemotherapy. The toxicity profile of erlotinib observed in this study was favourable. As is typical of erlotinib and other EGFR inhibitors, skin rash and diarrhoea were the most common side effects. For most patients, these side effects were tolerable, and for those with more severe rash, the use of minocycline enabled most patients to continue the study medication. In comparison, other EGFR inhibitors such as gefitinib and EKB-569 have shown some responses when given alone or in combination with chemotherapy (Fisher *et al*, 2004; Redlinger *et al*, 2004; Tejpar *et al*, 2004). However, most of these trials have used an untreated patient population.

This raises the question whether this and other targeted agent studies should be performed in patients previously untreated with chemotherapy. This does pose some additional challenges related to trial design and accrual. It also remains equally important to assess activity following chemotherapy, and ideally differences in activity and resistance based on prior therapy. Many novel agents have shown responses in treated and untreated populations (Kerr, 2004). The dose of targeted agents may be important for activity, however; the dose of erlotinib used in this study was based on phase I data (Hidalgo *et al*, 2001) and the toxicity from our study suggest that it would not be possible to increase the dose further in this patient population.

Based on the response data from our study and from other EGFR targeted therapies in development, it appears that only a portion of the population responds to targeted therapy. It may in fact be that other downstream signalling pathways are responsible for the resistance or that the response is influenced by some as yet undetermined pharmacodynamic or genetic factor unique to each patient. Therefore, there is a need to try to find markers to select patients who would benefit from targeted therapy. Three recent studies have demonstrated that gefitinib, another EGFR TK inhibitor, induced dramatic clinical responses in non-small-cell lung cancers with activating mutations within the EGFR kinase domain (Lynch *et al*, 2004; Paez *et al*, 2004; Pao *et al*, 2004). A biological marker or panel of markers that will select for response would allow future trials to screen the population being treated for those most likely to benefit. Other EGFR-targeted therapies such as the monoclonal antibody cetuximab (IMC-225) have shown antitumour activity in colorectal cancer (Saltz *et al*, 2001), suggesting that targeting this pathway is valid.

We conducted detailed correlative studies to investigate the pharmacokinetic and pharmacodynamic effects of erlotinib in tumour tissue. Practical problems encountered in getting matched tumour biopsies in all patients were medical contraindication of biopsy, logistic reasons preventing return for a second biopsy and difficulty analysing some of the tissue samples owing to degradation. This resulted in only 10 matched, biopsy pairs, of which eight contained tumour tissue. The fluorescence image analysis technique used was adapted from one previously used in our laboratory to show erlotinib pharamacodynamic effects in orthotopic xenografts of pancreatic cancer (Ng *et al*, 2002). Relative to immunohistochemistry, immunofluorescence imaging is more quantitative, since with appropriate background controls, the mean pixel values reflect the extent of primary antibody binding. To minimise the effects of observer bias, and to address the problems of intratumoral heterogeneity in marker expression, we used a scanning autostage to image the entire length of the core biopsies, and defined regions of tumour tissue using an anticytokeratin cocktail. Using this approach, we showed significant inhibition of EGFR and ERK in the post-treatment samples, consistent with EGFR signalling playing an important role in the downstream activation of the ERK pathway in colorectal cancer, and the successful disruption of this pathway by erlotinib.

Although there was no correlation found between s.d. and changes in correlative markers, this conclusion was based on a very small number of patients with serial tumour biopsies. Interestingly, we found no effects on the levels of phosphorylated PKB/Akt, which can also be activated by EGFR signalling via phosphatidylinositol 3′-kinase, the cyclin-dependent kinase inhibitor p27 or the proliferation marker Ki67. These findings have important implications for future clinical trials of EGFR inhibitors in colorectal cancer, as they imply that these agents are able to disrupt activation of ERK signalling, but that in itself is insufficient to produce major clinical responses. There were, however, many other markers that were not evaluated in this study because of limitations with tissue volume. Future studies need to be performed with EGFR TK inhibitors in order to help elucidate the exact markers that are affected in this downstream cascade.

The ability to predict the activity of an EGFR inhibitor on individual patients, or groups of patients based on the examination of molecular fingerprints from tumour specimens holds a lot of promise to move this investigational field forward. Unfortunately, an accurate method to predict response has not yet been established as was demonstrated by a study by Saltz *et al* looking at the treatment of colorectal cancer patients who express the EGF receptor with cetuximab. This trial demonstrated a lack of correlation between response and the degree of EGF receptor expression, which had been presumed to be one of the potential predictors for patient response to EGF receptor inhibitors (Saltz *et al*, 2002).

Recently, important results of erlotinib in patients with advanced non-small-cell lung cancer were published that demonstrated a survival advantage over best supportive care in lung cancer (Shepherd *et al*, 2005). This suggests that the administration of single agent EGFR TK inhibitors can result in significant clinical benefit and more work needs to be carried out on patient selection for targeted therapy trials based perhaps on a tumour genotype or tumour phenotype. Recent work by Tsao *et al* (2005) suggest that among patients with non-small-cell lung cancer receiving erlotinib, the presence of an EGFR mutation may predict for responsiveness to the agent; however, it was not indicative of a survival benefit. This implies that although we are gaining more insight into the possible indicators of activity, a greater understanding still need to be achieved.

## Figures and Tables

**Table 1:** Patient demographics for all 38 patients put on study

**Patient demographic**	**No. of patients (range)**
Age (median years)	63.9 (40.9, 78.1)
	
*Gender*	
Female : male	13 : 25
	
*ECOG PS*	
0 : 1	21 : 17
	
*No. of prior regimens*	
1 : 2 : 3	25 : 10 : 3
	
*Prior therapy*	
Adjuvant chemotherapy	16
Metastatic chemotherapy	32
Radiotherapy	12
	
*Target/nontarget sites*	
Median (range)	5 (1, 10)/2 (0, 5)
Nodes	19/15
Liver	28/15
Lung	10/16
Other	7/6

ECOG=Eastern Cooperative Oncology Group performance status.

**Table 2:** Grade 3 and 4 adverse events occurring in more that 2% of patients on this phase II study of erlotinib

**Grade 3/4 toxicities**	***n* (%) patients**	***n* (%) cycles**
Gr. 4 Constipation	2 (5)	2 (2)
Gr. 4 Abdominal pain/cramping	1 (3)	1 (1)
Gr. 4 Bone pain (left hip/right ankle)	1 (3)	1 (1)
Gr. 3 Diarrhoea	4 (11)	4 (4)
Gr. 3 Rash/desquamation	4 (11)	4 (4)
Gr. 3 Vomiting	3 (8)	3 (3)
Gr. 3 Pain	2 (5)	3 (3)
Gr. 3 Haematuria	2 (5)	4 (4)
Gr. 3 Hyperglycaemia	2 (5)	3 (3)
Gr. 3 Alkaline phosphatase	2 (5)	2 (2)
Gr. 3 Nausea	2 (5)	2 (2)
Gr. 3 Prothrombin time	2 (5)	2 (2)

**Table 3:** Most frequent toxicities of any grade experienced by patients on this phase II study of erlotinib

**All toxicities**	***n* (%) patients**	***n* (%) cycles**
Rash/desquamation	34 (90)	88 (79)
Lymphopenia	33 (87)	81 (72)
Diarrhoea	23 (61)	69 (62)
Hyperglycaemia	23 (61)	50 (45)
Fatigue/Lethargy	24 (63)	63 (56)
Haemoglobin	30 (79)	78 (70)
Alkaline phosphatase	24 (63)	52 (46)

**Table 4:** Tumour mean marker values for pre- and on-treatment performed by quantitative immunofluorescence (unit of measurement is the proportion of area in the epithelium positively stained for each individual marker)

	**Pretreatment median (range)**	**On-treatment median (range)**	**On- and pre-treat. median (range) difference**	**Simple *P*-value**
pEGFR	200 (62, 515)	123 (−41, 197)	125 (1, 318)	0.008
EGFR	159 (−10, 349)	200 (125, 552)	−122 (−303, 215)	0.31
pERK	280 (189, 590)	121 (−25, 512)	181 (35, 266)	0.008
ERK	415 (327, 765)	559 (172, 918)	−38 (−325, 277)	0.46
p27	927 (414, 1614)	771 (472, 1519)	−33 (−401, 631)	0.95
Ki-67/MIB-1	0.41 (0.26, 0.52)	0.31 (0.11, 0.46)	0.13 (−0.09, 0.26)	0.11
PKB/Akt	943 (566, 1215)	918 (694, 1118)	60 (−343, 364)	0.64

ERK=phospho-extracellular signal-regulated kinase; pEGFR=phospho-epidermal growth factor receptor tyrosine.

## References

[bib1] Carpenter G, Cohen S (1990) Epidermal growth factor. J Biol Chem 265: 7709–7712 2186024

[bib2] Cunningham D, Humblet Y, Siena S, Khayat D, Bleiberg H, Santoro A, Bets D, Mueser M, Harstrick A, Verslype C, Chau I, Van Cutsem E (2004) Cetuximab monotherapy and cetuximab plus irinotecan in irinotecan refractory metastatic colorectal cancer. N Engl J Med 351: 337–345 15269313 10.1056/NEJMoa033025

[bib3] Fisher GA, Kuo T, Cho CD, Halsey J, Jambalos CN, Schwartz EJ, Robert RV, Advani RH, Wakelee HA, Sikic BI (2004) A phase II study of gefitinib in combination with FOLFOX-4 (IFOX) in patients with metastatic colorectal cancer. Proc Am Soc Clin Oncol 22: 14S (abstr. 187)

[bib4] Giacchetti S, Perpoint B, Zidani R, Le Bail N, Faggiuolo R, Focan C, Chollet P, Llory JF, Letourneau Y, Coudert B, Bertheaut-Cvitkovic F, Larregain-Fournier D, Le Rol A, Walter S, Adam R, Misset JL, Levi F (2000) Phase III multicenter randomized trial of oxaliplatin added to chronomodulated fluorouracil–leucovorin as first-line treatment of metastatic colorectal cancer. J Clin Oncol 18: 136–147 10623704 10.1200/JCO.2000.18.1.136

[bib5] Gordon AN, Finkler N, Edwards RP, Garcia AA, Crozier M, Irwin DH, Barrett E (2005) Phase 2 evaluation of OSI-774, a potent oral antagonist of the EGFR-TK in patients with advanced ovarian cancer. Int J Gynecol Cancer 15: 785–792 16174225 10.1111/j.1525-1438.2005.00137.x

[bib6] Hidalgo M, Siu LL, Nemunaitis J, Rizzo J, Hammond LA, Takimoto C, Eckhardt SG, Tolcher A, Britten CD, Denis L, Ferrante K, Von Hoff DD, Silberman S, Rowinsky EK (2001) Phase I and pharmacologic study of OSI-774, an epidermal growth factor receptor tyrosine kinase inhibitor, in patients with advanced solid malignancies. J Clin Oncol 19: 3267–3279 11432895 10.1200/JCO.2001.19.13.3267

[bib7] Jasas KV, Fyles A, Elit L, Hoskins PJ, Biagi J, Dubuc-Lissoir J, Matthews S, Dancey J, Eisenhauer E, Oza AM (2004) Phase II study of erlotinib (OSI 774) in women with recurrent or metastatic endometrial cancer: NCIC CTG IND-148. Proc Am Soc Clin Oncol 22: 14S (abstr. 5019)

[bib8] Kerr DJ (2004) Targeting angiogenesis in cancer: clinical development of bevacizumab. Nat Clin Pract Oncol 1: 39–43 16264798 10.1038/ncponc0026

[bib9] Lynch TJ, Bell DW, Sordella R, Gurubhagavatula S, Okimoto RA, Brannigan BW, Harris PL, Haserlat SM, Supko JG, Haluska FG, Louis DN, Christiani DC, Settleman J, Haber DA (2004) Activating mutations in the epidermal growth factor receptor underlying responsiveness of non-small-cell lung cancer to gefitinib. N Engl J Med 350: 2129–2139 15118073 10.1056/NEJMoa040938

[bib10] Mayer A, Takimoto M, Fritz E, Schellander G, Kofler K, Ludwig H (1993) The prognostic significance of proliferating cell nuclear antigen, epidermal growth factor receptor, and mdr gene expression in colorectal cancer. Cancer 71: 2454–2460 8095852 10.1002/1097-0142(19930415)71:8<2454::aid-cncr2820710805>3.0.co;2-2

[bib11] Ng SS, Tsao MS, Nicklee T, Hedley DW (2002) Effects of the epidermal growth factor receptor inhibitor OS1-774, Tarceva, on downstream signaling pathways and apoptosis in human pancreatic adenocarcinoma. Mol Cancer Ther 1: 777–783 12492110

[bib12] Paez JG, Janne PA, Lee JC, Tracy S, Greulich H, Gabriel S, Herman P, Kaye FJ, Lindeman N, Boggon TJ, Naoki K, Sasaki H, Fujii Y, Eck MJ, Sellers WR, Johnson BE, Meyerson M (2004) EGFR mutations in lung cancer: correlation with clinical response to gefitinib therapy. Science 304: 1497–1500 15118125 10.1126/science.1099314

[bib13] Pao W, Miller V, Zakowski M, Doherty J, Politi K, Sarkaria I, Singh B, Heelan R, Rusch V, Fulton L, Mardis E, Kupfer D, Wilson R, Kris M, Varmus H (2004) EGF receptor gene mutations are common in lung cancers from ‘never smokers’ and are associated with sensitivity of tumors to gefitinib and erlotinib. Proc Natl Acad Sci USA 101: 13306–13311 15329413 10.1073/pnas.0405220101 PMC516528

[bib14] Perez-Soler R, Chachoua A, Hammond LA, Rowinsky EK, Huberman M, Karp D, Rigas J, Clark GM, Santabarbara P, Bonomi P (2004) phase II trial of the epidermal growth factor receptor tyrosine kinase inhibitor OSI-774, following platinum based chemotherapy, in patients with advanced, EGFR expressing, non-small cell lung cancer. J Clin Oncol 22: 3238–3247 15310767 10.1200/JCO.2004.11.057

[bib15] Philip P, Mahoney M, Thomas J, Pitot H, Donehower R, Kim G, Picus J, Fitch T, Geyer S, Erlichman C (2004) Phase II Trial of erlotinib (OSI-774) in patients with hepatocellular or biliary cancer. Proc Am Soc Clin Oncol 22: 14S (abstr. 4025) 10.1200/JCO.2005.14.696 16170173

[bib16] Pollack VA, Savage DM, Baker DA, Tsaparikos KE, Sloan DE, Moyer JD, Barbacci EG, Pustilnik LR, Smolarek TA, Davis JA, Vaidya MP, Arnold LD, Doty JL, Iwata KK, Morin MJ (1999) Inhibition of epidermal growth factor receptor associated tyrosine autophosphorylation in human carcinomas with CP-358,774: dynamics of receptor inhibition *in situ* and antitumor effects in athymic mice. J Pharmacol Exp Ther 291: 739–748 10525095

[bib17] Porebska I, Harlozinska A, Bojarowski T (2000) Expression of the tyrosine kinase activity growth factor receptors (EGFR, ERB B2, ERB B3) in colorectal adenocarcinomas and adenomas. Tumour Biol 21: 105–115 10686540 10.1159/000030116

[bib18] Redlinger M, Kramer A, Flaherty K, Sun W, Haller D, O'Dwyer PJ (2004) A phase II trial of gefitinib in combination with 5-FU/LV/irinotecan in patients with colorectal cancer. Proc Am Soc Clin Oncol 22: 14S (abstr. 3767)

[bib19] Ries LAG, Eisner MP, Kosary CL, Hankey BF, Miller BA, Clegg L, Mariotto A, Feuer EJ, Edwards BK (eds) SEER Cancer Statistics Review, 1975–2002. Bethesda, MD: National Cancer Institute; http://seer.cancer.gov/csr/1975_2002/, based on November 2004 SEER data submission, posted to the SEER web site 2005

[bib20] Rubin MS, Shin DM, Pasmantier M, Falcey J, Paulter V, Fetzer K, Waksal H, Mendelsohn J, Hong W (2000) Monoclonal antibody (MoAB) IMC-C225, an anti-epidermal growth factor receptor (EGFr), for patients (Pts) with EGFr-positive tumors refractory to or in relapse from previous therapeutic regimens. Proc Am Soc Clin Oncol 19: 474a (abstr. 1860)

[bib21] Salomon DS, Brandt R, Ciardiello F, Normanno N (1995) Epidermal growth factor-related peptides and their receptors in human malignancies. Crit Rev Oncol Hematol 19: 183–232 7612182 10.1016/1040-8428(94)00144-i

[bib22] Saltz L, Meropol NJ, Loehrer PJ, Waksal H, Needle MN, Mayer RJ (2002) Single agent IMC-C225 (Erbitux) has activity in CPT-11 refractory colorectal cancer that expresses the epidermal growth factor receptor. Proc Am Soc Clin Oncol 21: 127a (abstr. 504) 10.1200/JCO.2004.10.182 14993230

[bib23] Saltz L, Rubin M, Hochster H, Tchekmeydian NS, Waksal H, Needle M, LoBuglio A (2001) Cetuximab (IMC-225) plus irinotecan (CPT-11) is active in CPT-11 refractory colorectal cancer (CRC) that expresses epidermal growth factor receptor (EGFR). Proc Am Soc Clin Oncol 20: 3a (abstr. 7)

[bib24] Saltz LB, Cox JV, Blanke C, Rosen LS, Fehrenbacher L, Moore MJ, Maroun JA, Ackland SP, Locker PK, Pirotta N, Elfring GL, Miller LL (2000) Irinotecan plus fluorouracil and leucovorin for metastatic colorectal cancer. Irinotecan Study Group. N Engl J Med 343: 905–914 11006366 10.1056/NEJM200009283431302

[bib25] Shepherd FA, Rodrigues Pereira J, Ciuleanu T, Tan EH, Hirsh V, Thongprasert S, Campos D, Maoleekoonpiroj S, Smylie M, Martins R, van Kooten M, Dediu M, Findlay B, Tu D, Johnston D, Bezjak A, Clark G, Santabarbara P, Seymour L, National Cancer Institute of Canada Clinical Trials Group (2005) Erlotinib in previously treated non-small-cell lung cancer. N Engl J Med 353: 123–132 16014882 10.1056/NEJMoa050753

[bib26] Soulieres D, Senzer NN, Vokes EE, Hidalgo M, Agarwala SS, Siu LL (2004) Multicenter phase II study of erlotinib, an oral epidermal growth factor receptor tyrosine kinase inhibitor, in patients with recurrent or metastatic squamous cell cancer of the head and neck. J Clin Oncol 22: 77–85 14701768 10.1200/JCO.2004.06.075

[bib27] Tejpar S, Van Cutsem E, Gamelin E, Machover D, Soulie P, Ulusakarya A, Laurent S, Vauthier JM, Quinn S, Zacharchuk C (2004) Phase1/2a study of EKB-569, an irreversible inhibitor of epidermal growth factor receptor, in combination with 5-fluorouracil, leucovorin, and oxaliplatin (FOLFOX-4) in patients with advanced colorectal cancer (CRC). Proc Am Soc Clin Oncol 22: 14S (abstr. 3579)

[bib28] Therasse P, Arbuck SG, Eisenhauer EA, Wanders J, Kaplan RS, Rubinstein L, Verweij J, Van Glabbeke M, van Oosterom AT, Christian MC, Gwyther SG (2000) New guidelines to evaluate the response to treatment in solid tumors. European Organization for Research and Treatment of Cancer, National Cancer Institute of the United States, National Cancer Institute of Canada. J Natl Cancer Inst 92: 205–216 10655437 10.1093/jnci/92.3.205

[bib29] Tsao MS, Sakurada A, Cutz JC, Zhu CQ, Kamel-Reid S, Squire J, Lorimer I, Zhang T, Liu N, Daneshmand M, Marrano P, da Cunha Santos G, Lagarde A, Richardson F, Seymour L, Whitehead M, Ding K, Pater J, Shepherd FA (2005) Erlotinib in lung cancer – molecular and clinical predictors of outcome. N EngI J Med 353: 133–144 10.1056/NEJMoa050736 16014883

[bib30] Ullrich A, Schlessinger J (1990) Signal transduction by receptors with tyrosine kinase activity. Cell 61: 203–212 2158859 10.1016/0092-8674(90)90801-k

[bib31] Zee B, Melnychuk D, Dancey J, Eisenhauer E (1999) Multinomial phase II cancer trials incorporating response and early progression. J Biopharm Stat 9: 351–363 10379698 10.1081/BIP-100101181

[bib32] Zhang W, Siu L, Moore M, Chen EX (2005) Simultaneous determination of OSI-774 and its major metabolite OSI-420 in human plasma by using HPLC with UV detection. J Chromatogr B 814: 143–147 10.1016/j.jchromb.2004.10.016 15607718

